# Immunotherapy Using Dendritic Cells against Multiple Myeloma: How to Improve?

**DOI:** 10.1155/2012/397648

**Published:** 2012-03-15

**Authors:** Thanh-Nhan Nguyen-Pham, Yoon-Kyung Lee, Hyeoung-Joon Kim, Je-Jung Lee

**Affiliations:** ^1^Research Center for Cancer Immunotherapy, Chonnam National University Hwasun Hospital, Hwasun, Jeollanamdo 519-763, Republic of Korea; ^2^Department of Hematology-Oncology, Chonnam National University Hwasun Hospital, 160 Seoyangro, Hwasun, Jeollanamdo 519-763, Republic of Korea; ^3^Vaxcell-Bio Therapeutics, Hwasun, Jeollanamdo 519-763, Republic of Korea

## Abstract

Multiple myeloma (MM) is a good target disease in which one can apply cellular immunotherapy, which is based on the graft-versus-myeloma effect. This role of immune effector cells provides the framework for the development of immune-based therapeutic options that use antigen-presenting cells (APCs) with increased potency, such as dendritic cells (DCs), in MM. Current isolated idiotype (Id), myeloma cell lysates, myeloma dying cells, DC-myeloma hybrids, or DC transfected with tumor-derived RNA has been used for immunotherapy with DCs. Immunological inhibitory cytokines, such as TGF-**β**, IL-10, IL-6 and VEGF, which are produced from myeloma cells, can modulate antitumor host immune response, including the abrogation of DC function, by constitutive activation of STAT3. Therefore, even the immune responses have been observed in clinical trials, the clinical response was rarely improved following DC vaccinations in MM patients. We are going to discuss how to improve the efficacy of DC vaccination in MM.

## 1. Introduction

Multiple myeloma (MM) is a clonal B cell malignant disease that is characterized by the proliferation of plasma cells in the bone marrow (BM) in association with monoclonal protein in the serum and/or urine, immune paresis, skeletal destruction, renal dysfunction, anemia, hypercalcemia and lytic bone diseases [1, 2]. Although the introduction of conventional chemotherapy, high-dose therapy with hematopoietic stem cell transplantation (HSCT), and the development of novel molecular target agents has resulted in a marked improvement in overall survival, the disease still remains incurable [3, 4]. Alternative approaches are clearly needed to prolong the disease-free survival, as well as the overall survival of patients with MM. To prolong the survival of patients with MM who are undergoing allogeneic HSCT, donor lymphocyte infusion can be used successfully as a salvage therapy, which is based on the graft-versus-myeloma effect in some cases of MM that relapse after allogeneic HSCT [5–7]. This role of immune effector cells provides the framework for the development of immune-based therapeutic options that use antigen-presenting cells (APCs) with increased potency, such as dendritic cells (DCs), in MM [6, 7].

DCs are the most potent APCs for initiating cellular immune responses through the stimulation of naive T cells. Immature DCs are good at antigen uptake and processing, but for a stimulatory T-cell response they must mature to become fully activated DCs, which express high levels of cell surface-related major histocompatibility complex- (MHC-) antigen and costimulatory molecules. Because of their ability to stimulate T cells, DCs act as a link in antitumor immune responses between innate immunity and adaptive immunity [8]. These DCs play a central role in various immunotherapy protocols by generation of cytotoxic T lymphocytes (CTLs) [9]. DC-based vaccines have become the most attractive tool for cancer immunotherapy and have been used in the treatment of more than 20 malignancies, most commonly melanoma, renal cell carcinoma, prostate cancer, and colorectal carcinoma [10, 11]. In MM, cellular immunotherapy using DCs is emerging as a useful immunotherapeutic modality to treat MM [10]. Since tumor antigen-loaded DCs are expected to be able to stimulate tumor-specific CTLs and to overcome T cell tolerance in tumor patients, the development of DC vaccines that can consistently eliminate minimal residual neoplastic disease remains an important goal in the field of tumor immunology [12].

## 2. Current DC Therapy in MM

MM is believed to induce immunoparesis that interferes with DC function, which diminishes the effective antitumor immune responses in these patients. Usually, *ex vivo* DCs are generated from circulating blood precursors (i.e., monocytes) or bone marrow progenitor cells and are educated with tumor antigens prior to vaccination to patients. *Ex vivo* generated DCs can be loaded with myeloma-associated antigens as vaccines for patients with MM. The use of immature DCs or mature DCs, the way to induce DC maturation, types of tumor antigens, the techniques to load tumor antigens to DCs, routes of administration, and dosing schedules are being investigated [13].

### 2.1. Idiotype-Pulsed DCs

Immunoglobulin Idiotype (Id) is a tumor-specific antigen can be defined that each B cell tumor clone produces. Id can be readily isolated from the plasma of MM patients [14]. The Id protein has been used for immunotherapy both *in vitro* and *in vivo* in MM and has demonstrated a successful response in follicular lymphoma and a unique expression of Id on the malignant B cell clone [15, 16]. Id vaccination could induce both antibody and Id-specific T cells including CD4^+^ T cell and CD8^+^ T cell response by the presentation of Id protein on MHC class I and II of professional APCs, such as DCs. Id-specific CTL lines could be generated that killed autologous primary myeloma cells *in vitro*, and killing activity was induced by only MHC class I restricted [17], while in the other report both class I and class II restriction was observed [18]. Autologous DCs that were generated from MM patients have been shown to efficiently endocytose different classes of Id protein, and autologous Id-specific CTLs lines containing both CD4^+^ and CD8^+^ T cells that were generated by Id-pulsed DCs significantly recognized and killed the autologous primary myeloma cells *in vitro* [18, 19]. Until now, the various studies of DC-based Id vaccination in MM have been reported [20–27]. Although Id-specific CTLs and immune response could be induced in some patients, clinical responses have been observed rarely in few patients after vaccination [22]. To improve the effectiveness of DC vaccination, the Id-pulsed DCs were vaccinated in combination with KLH or cytokine IL-2 in MM patients [21, 23, 26]. However, even both cellular and antibody responses have been observed, the clinical response also was not improvement following vaccinations. The reasons for these results may be attributed mainly to the Id protein as a weak antigen, and the use of immature DCs in some studies [20, 28, 29].

### 2.2. Myeloma-Associated Antigens-Loaded DC

Tumor-associated antigens (TAAs) have been identified in many tumor types including solid tumors and hematological malignancies. The highly specific TAAs overexpress in increasing amounts in malignant cells were the greatest potential for clinically useful assays. A variety of myeloma-associated antigens have been identified in MM patients, which possibility provides an immune response by DC-based vaccine. T cells from myeloma patients can recognize a variety of TAAs, which suggesting that the T cell has the capacity to kill myeloma cells selectively if these clonal populations can be activated and expanded effectively by a potent TAA. Many potential TAAs in MM have been investigated including polymorphic epithelial mucin (MUC1), human telomerase reverse transcriptase (hTERT), PRAME, HM1.24, SP17, Wilms' tumor I (WTI), Dickkopf-1 (DKK1), or member of cancer germ-like family (MAGE, GAGE, BAGE, LAGE, NY-ESO-1) [30–35]. Among the various TAAs, some have been tested as peptide vaccines and only a few of them has been tested *in vitro* to induce TAA-specific CTLs response via loading the potent TAA to DCs in MM. The first TAAs pulsed with DCs in MM was MUC1, which was expressed on all of MM cell lines and primary myeloma cells and in sera of MM patients. Vaccination with MUC1 antigen has not been studied in MM patients, but MUC1-specific CTLs that were induced *in vitro* using peptide-pulsed DCs or plasma cell RNA-loaded DCs efficiently killed not only target cells pulsed with the antigenic peptide but also MM cells [31, 36]. NY-ESO-1 is the most immunogenic of the cancer testis antigens, which are expressed in a variety of tumors, while their presence in normal tissue is limited to the testis and placenta [35]. In MM, expression of NY-ESO-1 has been correlated with more advanced disease [37]. Spontaneous humoral and CD8^+^ T cell-mediated responses to NY-ESO-1 have been identified in patients with advanced disease [35, 37]. The *in vitro* monocyte-derived DCs transduced with the PTD-NY-ESO-1 protein can induce CD8^+^ cellular antitumor immunity superior to that achieved with NY-ESO-1 protein alone [30]. Sperm protein 17 (Sp17), the other immunogenic TAA, has been used as a tumor antigen to load into DCs. Sp17-specific HLA class I restricted CTLs were successfully generated by DCs that have been loaded with a recombinant Sp17 protein and the CTLs were able to kill autologous tumor cells that expressed Sp17 [38, 39]. The over-expression of hTERT on MM compared to normal cells indicated that this telomerase could be used as tumor antigen to induce antitumor immune responses. hTERT was capable of triggering antitumor CTL responses and kill hTERT^+^ tumor cells [40]. Recently, the CTLs that were stimulated by hTERT- and MUC1-derived nonapeptides loaded DCs were successfully able to kill myeloma cell line [41]. DKK1, a novel protein that is not expressed in most normal tissues but is expressed in almost myeloma cells, could be a potentially important antigenic target for antimyeloma immunotherapy. DKK1-specific CTLs that were generated by DCs pulsed with DKK1 peptides were specifically lysed autologous primary myeloma cells and DKK1-positive cell line [34]. In general, TAAs could be a major interest in immunotherapy in MM. Taken together, the data support DC immunotherapy with TAAs as being a promising immunotherapy to support to clinical trials in MM.

## 3. Whole Tumor Antigen-Loaded DC

An alternative to Id protein- or TAA-based immunotherapy in MM is to use other tumor antigens that derived from whole tumor preparation to improve the efficacy of the DC vaccination in patients with MM. DCs loaded with antigens derived from whole tumor cells can improve the antitumor response and that limits the risk for immunological escape. There have been increasing reports of these alternative approaches, such as DCs pulsed with myeloma lysates [42–44], DCs pulsed with myeloma apoptotic bodies [43, 45, 46], DCs transfected with myeloma-derived RNA [36], DCs pulsed with myeloma-derived heat shock protein (HSP) gp96 [47, 48], or DC-myeloma cell hybrids [49–51]. These techniques have the advantage of allowing the presentation of multiple epitopes to MHC on DCs, therefore can induce polyclonal T-cell response from many potentially unknown TAAs and reduce the probability of immune escape by single TAA. DCs loaded with myeloma cell lysates demonstrated much stronger cytotoxicity against autologous plasma cells than did those by Id protein-pulsed DCs, which suggested the superiority of the myeloma cell itself as a source of a tumor antigen compared with the Id protein [44]. In other myeloma model, DCs pulsed with purified and optimized myeloma cell lysate were shown to generate CTLs that killed autologous tumor cells but not against mismatch HLA cell lines or K562 cell lines *in vitro* [43]. The apoptotic bodies derived from either myeloma cell lines or patient's myeloma cells also have been used as tumor antigen to loading with DCs. Interestingly, apoptotic bodies were shown to be more effective than cell lysate at inducing CTLs against autologous myeloma cells [42]. Heat shock proteins (HSPs) are a class of functionally related proteins whose expression is increased when cells are exposed to elevated temperatures or other stress. Tumor-derived HSPs, such as HSP70 and gp96, are immunogenic and potent in stimulating the generation of tumor-specific CTLs. The myeloma-derived gp96 loaded DCs were used to generate tumor-specific CTLs that were able to lyse myeloma tumor cells but not normal blood cells in a MHC class I restricted manner [47, 48]. In other way, the fusions of autologous DCs with patient-derived tumor cells have been developed. Fusion cells can stimulate both helper and cytotoxic T-cell responses through the presentation of internalized and newly synthesized antigens [51]. In mouse MM models, vaccination with DCs fused with either plasmacytoma cells or tumor cells that were genetically modified to express CD40L resulted in eradication of disease in tumor-bearing animal and protective against subsequent tumor challenge in animals [49, 50]. In general, the production of DC vaccine by using whole tumor antigens has become promising in order to induce immunotherapy against MM.

## 4. DC-Based Vaccine Clinical Trials

Clinical trials of DC-based vaccine for MM have been restricted until now. The trial protocol and responses are summarized in Table 1. ALmost of the clinical trials were related with using Id-pulsed DC alone or in combination with adjuvant such as cytokines or KLH. In the decade after the first DC-based Id vaccination was started at Stanford University, the results of clinical trials were limited. In general, the majority of clinical trials conducted using Id-pulsed DCs showed immune responses. However, the clinical responses were unsatisfactory, mainly due to the poor immunogenicity of the Id protein. More recent results demonstrated improved clinical response by DC-based Id vaccination [26, 27]. Therefore, DC-based Id vaccination is going to a possible way to induce the specific T cell responses in myeloma patients. Further trials with increasing numbers of patients are needed to increase the rate of responses.

Most recently, phase I study was undertaken, in which patients with MM were vaccinated with an autologous DC/tumor cell fusion in combination with GM-CSF administration on the day of DC vaccination [52]. Vaccine generation was successful in 17 of the 18 patients. The expansion of circulating CD4^+^ and CD8^+^ T cells reactive with autologous myeloma cells in 11 of 15 evaluable patients were detected. A majority of patients (11 of 16) with advanced disease demonstrated disease stabilization, with three patients showing ongoing stable disease at 12, 25, and 41 months. Interestingly, antibody response against some TAAs, such as regulators of G-protein signaling 19 (RGS19), HSP90, BRCA1-associated protein (BRAP), was also detected. So, vaccination with DC/MM fusions was feasible and may provide a new source of DC-based vaccines for the development of immunotherapy against MM.

A commercial product is currently being tested in phase III trial (Mylovenge, Dendreon Corp, Seattle, WA, USA). Mylovenge (APC8020) is conducted by pulsing autologous DCs with the patient's Id. A recent report of this commercial product showed that the long-term survival of those receiving the vaccine compared to all other patients with MM who underwent autologous HSCT [53]. This approach needs further testing in phase III trial to confirm the clinical response and define the role of this DC vaccine in MM. We are also conducting phase I/II clinical trial using type-1-polarized DCs loading with tumor antigens derived either from allogeneic myeloma cell line or patient's autologous-/allogeneic-myeloma cells in combination with chemotherapy in patients with MM after autologous HSCT.

## 5. How to Improve DC Vaccination in MM?

During recent decades, cancer immunotherapy using DC-based vaccines has been used as therapeutic in patients with cancer including MM patients, however, while a few number of patients can really induce tumor regressions, one of the most common responses of the current DC vaccination is only a demonstration of antigen-specific immune responses, but no evidence of tumor regression. This unexpectation provides the new strategy for the treatment of cancer in which the intrinsic abilities of the immune system response to the DC vaccine has been modified to enhance the efficacy of vaccination. Several studies indicate that the immune system of cancer patients can recognize and kill tumors; however, some cancer patient cannot induce the immune response against tumor. In particularly, *ex vivo* DCs are usually generated from cancer patients, however, patients with cancer including MM have basically dysfunctional DCs [54–57]. DC function is mainly affected by the microenvironment in which they can stimulate immune response [58]. The present of several immunosuppressive factors in tumor microenvironment including the high production of inhibitory cytokines (interleukin-(IL-) 10, transforming growth factor beta (TGF-*β*), vascular endothelial growth factor (VEGF), and IL-6), the activation of STAT3, the expansion of Treg cells, and the significant suppressive effect of MDSC has been investigated [55–57, 59–64]. Therefore, the recent new idea now is how to improve the efficacy of DC vaccine to increase the effectiveness of vaccination against tumors.

**Figure 1:**
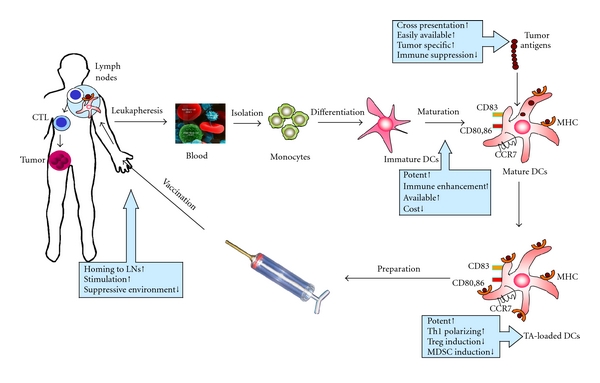
Key points to improve DC vaccination in cancer patients. CTL: cytotoxic T lymphocyte; DCs: dendritic cells; TA: tumor antigen; LNs: lymph nodes; Treg: regulatory T cell; MDSC: myeloid-derived suppressor cell.

For improving clinical outcomes using DC-based immunotherapy, there have been increasing reports of alternative approaches, such as better cytokine combinations to enhance DC function, effective tumor antigens to induce specific CTLs, or modifying signal transcriptions to overcome defective DC function. Our experience in the DC research field has revealed several key points to improve DC vaccination in cancer patients including MM (Figure 1).

### 5.1. Enhancing the Maturation and Activation of DCs by Th1 Polarizing Cytokines

For effective induction of tumor-specific immune responses in the field of DC vaccination, the DCs should have potency to stimulate T cells, to produce high levels of Th1-polarized cytokines (IL-12p70), to trigger Th1 polarizing capacity, and to migrate through lymphatic vessels to interact with T cells. The initial success of the therapeutic vaccines involving immature or partially-mature “first-generation” DCs has been reported [65]. However, such DCs express suboptimal levels of costimulatory molecules, and constitute a weaker immunogen than the subsequently implemented mature DCs, constituting the “second generation” of clinically applied DCs (sDCs). sDC vaccines induced by the IL-1*β*/TNF-*α*/IL-6/prostaglandin E_2_ (PGE_2_) cytokine cocktail have been developed [66]. Such DCs are fully mature DCs with high expression of costimulatory molecules, high expression of CCR7, and high migratory responsiveness to LN-associated chemokines; they have been widely tested in clinical trials. However, to date, the sDC vaccines have limitations that include the mediation of Th2 polarization, promotion of DC secretion of the immunosuppressive cytokine IL-10, inability to induce effectively the Th1-type response (because PGE_2_ abolishes the secretion of IL-12p70), and high activity of such DCs in activating Treg cells [67–70].

Several investigators, including our group, have tried to develop the potent DCs for inducing effective tumor-specific immune responses. In an attempt to increase DC potency using cytokine combinations, *α*-type-1-polarized DCs (*α*DC1s) that are induced to mature using the *α*DC1-inducing cytokine cocktail IL-1*β*, TNF-*α*, IFN-*α*, IFN-*γ*, and polyinosinic: polycytidylic acid [poly(I:C)] has been developed to generate strong functional CTLs in several diseases, on average 20-fold higher compared to sDCs [71, 72]. Recently, we successfully generated *α*DC1s from a patient with MM with high expression of costimulatory molecules, significant production of IL-12p70, and potent generation of myeloma-specific CTLs [43, 46]. Such a novel appropriate strategy provides a way to improve the potency of *ex vivo* generated DCs for cancer therapy.

### 5.2. Enhance the Maturation and Activation of DCs by Natural Product as TLR Signaling

Ursolic acid (URC) is isolated from Uncaria rhynchophylla and phytochemically classified as triterpene. Triterpene compounds have been identified as a unique class of natural products possessing diverse biological activities. Recently, we have reported that URC activates human DCs in a fashion that favors Th1 polarization via the activation of IL-12p70 dependent on TLR2 and/or TLR4 and induces the production of IFN-*γ* by CD4^+^ naïve T cells [73]. In addition, the combination of URC and IFN-*γ* enhance the activation of DCs, namely, the enhancement of Th1 cells polarization that induced by IFN-*γ* depends on the activation of IL-12p70 and independent on TLR4 [74]. The potential of natural product to enhance DC maturation and activation has important implications for the use of DCs as cancer vaccines.

### 5.3. Enhance the Cross-Presentation of DCs by Tumor Associated Antigens

As described above, the results of immunotherapy with Id-pulsed DCs have been unsatisfying. The use of TAA can induce the higher immune response compared to Id. Although a single TAA has the possibility to induce the antitumor immune responses against MM, tumors may escape immune recognition by downregulating expression of a particular antigen. However, TAA can induce autoimmunity. Several TAAs have been detected in normal tissues. In addition, only a small number of tumor samples from MM patients showed a similar level of TAA expressing, limiting its usefulness for using TAA in MM. Therefore, to overcome the effect of TAAs-based immunotherapy, our group tries to use other tumor antigens that improve the cross-presentation of the DC vaccination in patients with MM.

 The selected antigen should possess the best characteristics to induce high cross-presentation, be tumor specific, be easily available, and be unable to induce immune suppression. Whole tumor antigens is the best tumor antigen, which has been selected by many investigator including myeloma cell lysates [42–44], apoptotic bodies from myeloma cell line [43, 45, 46]. In practical terms, there are a number of patients with MM, who have less than 50% of myeloma cells in the bone marrow at the time of diagnosis or during progression of the disease. When mononuclear cells from the bone marrow are used as a source of tumor antigens, there is the potential of contamination with normal cells, especially lymphocytes. Thus, it is necessary to use purified and optimized myeloma cells, if possible, as a source of tumor antigen for the generation of myeloma-specific CTLs stimulated by DCs [43]. We have shown that the function of the DCs was affected by the concentration of myeloma cell lysates (i.e., higher concentrations of lysates suppress T cell stimulatory capacities more than lower concentration of lysates). Also, the optimization of the lysate concentration did not demonstrate any inferiority in functions, such as T cell stimulatory capacities and cytotoxicities, of the DCs compared with other antigens, such as apoptotic bodies of myeloma cells or formalin-fixed myeloma cells. CTLs that were generated by purified and optimized myeloma cell lysates pulsed with DCs demonstrated much stronger cytotoxicity against autologous plasma cells. These findings indicate that it is important to optimize the concentration of myeloma cell lysates that were loaded onto DCs to potentiate their function.

The use of whole tumor cells, instead of single antigens, may help to enhance antitumor effects but target multiple tumor variants and counteract tumor immune evasion. However, it is impractical to obtain sufficient amounts of purified autologous myeloma cells for tumor antigens in the clinical setting of patients with MM. As an alternative source of tumor-relevant antigens, allogeneic tumor cells or established cancer cell lines have been used to overcome this limitation in various tumors [43, 46, 75, 76]. Allogeneic myeloma cell lines used as universal tumor antigens could substitute for an original tumor cell collection and make the culture of tumor cells easier. In clinical practice, allogeneic myeloma cell lines might be an effective source of universal tumor antigen that could be used to load DCs for the generation of myeloma-specific CTLs in MM patients. Tumor antigens that derived from irradiated allogeneic myeloma cell line when loaded with DCs could generate myeloma-specific CTLs against autologous myeloma cells in patients with MM [45, 46]. The success of using an allogeneic myeloma cell line as tumor antigen led to the possibility that allogeneic myeloma cells could be also used as a viable source of tumor antigen in the context of appropriate major MHC alleles to autologous CTLs. We investigated the possibility of DC therapy using autologous DC loaded with apoptotic allogeneic myeloma cells from the matched monoclonal subtype of myeloma patients and showed that the CTL generated by these tumor antigens loaded DCs could generate myeloma-specific CTLs against autologous myeloma cells in patients with MM [77]. These findings suggested that allogeneic myeloma cell lines and the allogeneic matching monoclonal immunoglobulin subtype of myeloma is the effective tumor antigen capable of inducing functional CTLs against patients' own tumor cells.

### 5.4. Blocking the Immunosuppressive Activity

The suppressive effects of tumor cells during DC generation have been explained previously by the ability of the tumor microenvironment to suppress DC differentiation [60, 78]. In addition, patients with MM have DCs that are functionally defective, evidenced by the decreased number of circulating precursors of DCs as well as impaired T-cell stimulatory capacity [55–57]. DCs in MM patients are a target of tumor-associated suppressive factors, such as IL-10, TGF-*β*, VEGF, and IL-6, resulting in their aberrant functions and impaired development of effector functions in tumor-specific lymphocytes [55, 56]. These factors can influence the activation of STAT3 and extracellular signal-regulated kinase (ERK) phosphorylation, resulting in hyperactivation of STAT3 and ERK, which may be responsible for defective DC differentiation [60, 79]. In addition to generation of potent and specific tumor antigen-loaded DCs for vaccination, alternative methods have attempted to restore defective DC function and to enhance DC function in MM. Enhanced immune-mediated antitumor effects of DCs have been reported following the inhibition of the janus-activated kinase 2 (JAK2)/STAT3 pathway [80–82], inhibition of p38 or activation of the MEK/ERK or mitogen-activated protein kinase (MAPK) pathways, and neutralization of IL-6 [83]. Recently, we reported that the inhibitory factors and abnormal signaling pathways of DCs during maturation with tumor antigen might be responsible for the defective activity of DCs in MM and suggested that the way to overcome these abnormalities is by neutralizing the signaling that would lead to a suppressed immune response [84]. More recently, we are developing of the strategies that recovering dysfunction of DCs caused from loading tumor antigen through the treatment of a combination of the selective JAK/STAT3 signaling pathway inhibitor (JSI-124) and the proteasome inhibitor (Bortezomib) onto myeloma cells (unpublished data). We reported that pretreatment of myeloma cells with combination of JSI-124 and bortezomib can recover DC dysfunction from loading the dying myeloma cells through the upregulation of Hsp90 and the downregulation of STAT3 phosphorylation and inhibitory cytokines production, and these DCs can generate to potent myeloma-specific CTLs.

### 5.5. Natural Killer (NK) Cells and Helper Functions during Induction of Type 1 Immunity by DCs

The other strategy to induce potent DCs from patients with MM was the use of a “helper” cell to promote type 1 polarization of DCs. NK cells are rapidly homing to the sites of infection and control the immune response in viral infections. Indeed, it has been demonstrated that NK cells play a major immunoregulatory role in the development of a protective T-cell-mediated immunity against intracellular pathogens and cancer [85–87]. Such “helper” activity of NK cells is at least partially mediated by the functional modulation of DCs, the phenomenon depending on the production of IFN-*γ* and TNF-*α* by activated NK cells [85–87], and associated with enhanced cross-presentation of tumor antigens and the induction of Th1 and CTL responses [45, 88, 89]. Recent data from our and other groups demonstrate that such NK-DC interaction promotes the subsequent induction of tumor-specific responses of CD4^+^ and CD8^+^ T cells, allowing NK cells to act as “helper” cells in the development of the type 1 DCs in responses against cancer [45, 88, 89]. Resting NK cells that are activated in the presence of TLR agonist, IL-2, and IFN-*α* can induce DCs from patients with MM maturation and enhance IL-12p70 production *in vitro*. These potent DCs can be developed to generate strong functional CTLs against myeloma cells compared to sDCs [45].

### 5.6. Treg Cells and MDSC Regulation

Therapeutic DC vaccines against cancer not only need to be highly effective in inducing the expansion of tumor-specific T cells, but they also need to avoid interaction and induction of Tregs. However, MM induces immune paresis [54]. Tumors are able to escape immune surveillance by down-regulation of immune responses as well as through the production of immunosuppressive cytokines by the tumor cells or by activation of suppressor cells such as regulatory T cells (Treg) and myeloid-derived suppressor cells (MDSCs) [90]. Dysregulation of natural CD4^+^CD25^+^ T regulatory (Treg) in MM has been reported [91]. Tregs are a group of immunosuppressive T cells that have been implicated in the suppression of tumor immunity [92]. A higher number of Tregs were reported in myeloma capable of suppressive activity at T-cell stimulation [61]. Recently, the discovery of MDSCs revealed these cells as potent suppressors of tumor immunity and, therefore, a significant impediment to cancer immunotherapy [63]. MDSCs can suppress the activation of T cells, B cells, NK cells and NKT cells. In contrast, MDSCs can enhance the induction of Tregs [64]. Recently, a human study reported that the proportion of CD4^+^FoxP3^+^ Treg cells and CD14^+^HLA-DR^−/low^ MDSC was increased in patients with MM at diagnosis was described [62]. These cells were functionally intact as they were able to inhibit proliferation of both CD4 and CD8 T cells illustrating that this cell fraction is also distorted in patients with MM [62].

The type-1-polarized DCs were demonstrated to suppress the secretion of CCL22 (Treg and Th2 type attracting chemokines), enhance the secretion of CCL5 and CXCL10 (Th1 and effector T-cell-attracting chemokines), and suppress the induction of Tregs compared to sDCs or PGE_2_-matured DCs [93]. In addition, to enhance the antitumor effectiveness of DC-based vaccines in preclinical *in vivo* mouse models, we have developed several models of combination therapy of DCs with an immunomodulatory drugs, such as cyclophosphamide or lenalidomide. Cyclophosphamide is frequently used to enhance or augment the antitumor effects in cancer immunotherapy [94]. The possible effect of cyclophosphamide to enhance the antitumor efficacy of DC vaccine may be due to the increasing proportion of IFN-*γ* secreting lymphocytes in combination with the suppressing proportion of CD4^+^CD25^+^FoxP3^+^ Treg cells in tumor-bearing mice [95]. The result of a clinical trial using allogeneic DC vaccine combined with low-dose cyclophosphamide has revealed that the combination therapy could induce stronger antitumor response compared with DC vaccine alone [96]. Recently, we developed a combination therapy in mouse cancer model which showed that a single administration of low-dose cyclophosphamide before the first DC vaccination augmented the antitumor effects of DC vaccine to eradicate tumor completely and consequently prolonged the survival of vaccinated mice [89]. Lenalidomide is a potent anti-myeloma drug which the activity are related with immunomodulatory properties. Lenalidomide inhibits Treg expansion and FoxP3 expression on cancer patients [54]. Our results show that the reduction of suppressor cells including Treg and MDSC in spleens of lenalidomide vaccinated mice in MM model (unpublished data). Therefore, the combination of DCs with chemotherapy, especially immunomodulatory drugs, could regulate and inhibit the expansion of immunosuppressor cells and significantly improve the antitumor effects.

### 5.7. Regulation the Migratory Pattern of DCs

DCs generated *in vitro* for vaccination protocols that can target a local lymph node are highly sought, but difficult to achieve in practice. Type-1-polarized DCs, with higher levels of IL-12p70 and potent CTL generation targeting, are, however, limited by their migratory capacity to primary lymph organs due to the relatively lower expression of CCR7 compared to sDCs. We recently reported on the nature of the enhancement of the migratory phenotype of DCs. The first important mediator in the mobilization of DCs to lymph nodes is CCR7. However, upregulation of CCR7 alone by DCs is insufficient to drive DC migration toward CCL19 and CCL21. Up-regulation of CD38 and downregulation of CD74 regulate DC migration *in vitro* and *in vivo* [97, 98]. By regulating CD38, CD74, and CCR7 expression on DCs, types I and II IFNs have synergistic effects in the presence of TLR agonists on the regulation of DC migration and may provide a novel approach to improving vaccination efficacy [99].

## 6. Can Cellular Immunotherapeutic Methods Improve MM?

In terms of treatment strategies in MM, the widespread use of the novel therapies, such as thalidomide, bortezomib and lenalidomide, has now significantly improved the prognosis, and outcome for patients [100]. The use of these novel therapies in the primary setting together with conventional chemotherapeutics into early treatment has driven most of the benefit. However, relapsed and refractory disease remains an area of challenge, where once prior therapy with immunomodulatory agents and proteasome inhibition has failed, the prognosis remains very poor. An important challenge that therefore emerges is a risk-adapted approach to MM therapy [101]. The application of some novel antitumor agents reveals the new option for high-risk MM. Autologous and allogeneic stem cell transplantation also still remains the important therapeutic modality for these patients.

Several studies investigated the point to an inherent immune system dysregulation that cause the complication of immunotherapeutic strategies for MM. The dysregulation in immune cells, the overproduction of immunosuppressive cytokines, and the proliferation of regulatory T cells and MDSCs have been associated with many defects in the host immune system of patients with MM, particularly in the advanced MM patients [54]. Adoptive transfer of T cells and NK cells may represent a new immunotherapy for multiple myeloma. One strategy to improve responses to vaccination involves combining active vaccination with adoptive T cell transfer [102]. Adoptive transfer of activated NK cells in conjunction with IL-2 to myeloma-bearing mice resulted in prolonged survival compared with treatment with either IL-2 or activated NK cells alone and the antimyeloma effect was more potent with a higher dose of NK cells [103].

**Figure 2:**
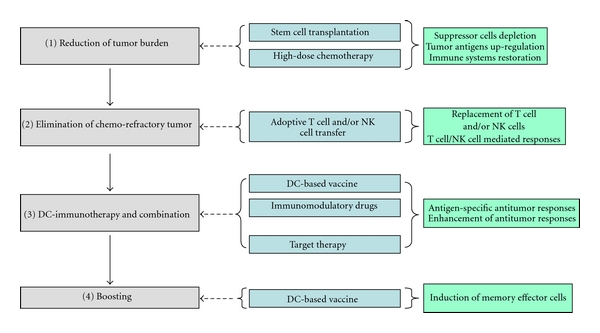
Current suggestion of DC-based vaccines for patients with MM. (1) Vaccination requires the restoration immune system and the tumor burden is low; (2) new T-cell repertoire induction and elimination of relapse/refractory disease; (3) DC vaccination in alone or combination; (4) boosting the antitumor immune responses.

For the DC-based vaccine, several clinical trials applied to the seeing of MM patients after ASCT and these approaches may reasonable to increase therapeutic effect of DC-based vaccine in term of minimal residual disease [20, 22–24, 28]. Furthermore, recent study has shown that the immune competence of MM patients can be restored following high dose chemotherapy and ASCT by a combination of vaccination and adoptive T-cell therapy [102]. Practically, patients with refractory and relapsed MM may be not good candidates to apply the DC-based vaccine, but combination approach using DC-based vaccine to reduce tumor cells and immune modulation agents, such as lenalidomide and low-dose cyclophosphamide, to overcome tumor microenvironment will be helpful to improve the disease status. The time point of DC-immunotherapy application was described in Figure 2.

## 7. Conclusion

**Figure 3:**
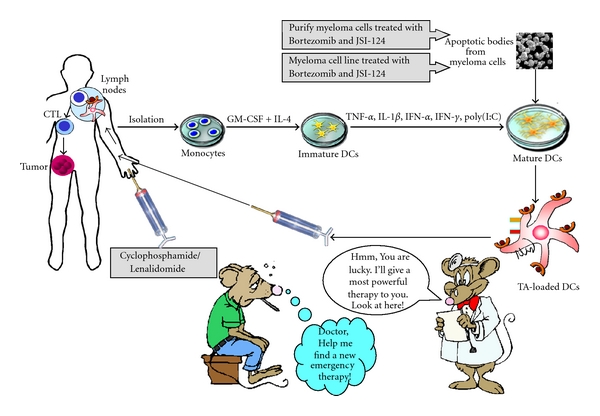
Generation of DC-based vaccines from patients with MM. Isolated monocytes from peripheral blood of patients are cultured with GM-CSF and IL-4 to produce immature DCs. Immature DCs were matured with *α*-polarizing cytokines cocktail to generate *α*-type 1-polarized DCs and were loaded with apoptotic bodies from myeloma cells or myeloma cell line which were induced in the presence of bortezomib and JSI-124. Tumor antigens-loaded DCs were then injected into patients in combination with either cyclophosphamide or lenalidomide to induce strong immune responses against the tumor.

Despite their relative limitations, the data from recent clinical studies have suggested that DC-based vaccine may be a potential therapy in inducing the rate of tumor responses and prolonging the survival of patients with MM. In an attempt to increase DC-based potency and improve immune responses following vaccination, further investigations of additional tools to identify the alternative tumor antigens uniquely or specifically expressed on myeloma cells are needed, to recover or restore the dysfunction of DCs in MM patients, to induce T cells with the desirable effector functions rather than regulatory functions, to migrate into lymph nodes to stimulate T cells, and to clarify the ability of tumor-specific CTLs to recognize and kill tumor cells. In our expectation, type-1-polarized DCs can be developed to generate strong functional CTLs. The allogeneic myeloma cell lines or allogeneic myeloma cells might be an effective source of universal tumor antigen that could be used to load to the DC1s for the successful generation of myeloma-specific CTLs. Eventually, the combination therapy, in which a DC vaccine is combined with either alternative therapy including chemotherapy, radiation therapy, molecular target therapy or other immunotherapy (adoptive therapy, NK cells therapy), or with adjuvant, will provide vigorous and maintained immune responses with the benefit clinical efficacy. The most promising DC-based vaccine in patients with MM was described in Figure 3.

## Figures and Tables

**Table 1:** Summary of clinical trials of DC-based vaccine for MM.

Author	DC type	TA	Adjuvant	Immune responses	Clinical responses
Liso et al.	imDC	Id	±KLH	4/24 Id-specific	17/26 SD

Lim et al.	imMo-DC	Id	KLH	5/6 Id-specific; 2/6 Id-specific IFN-*γ*; 3/6 increase in Id-specific CTL frequency	6/6 PD

Reichardt et al.	imDC	Id	none	2/12 Id-specific proliferation; 1/3 Id-specific CTL	2 relapse; 8/10 PD; 2/10 SD

Titzer et al.	CD34-DC	Id	none	4/10 Id-specific T cell proliferation; 1/10 decreased BM plasmacytosis	1/10 SD; 9/10 PD

Cull et al.	imMo-DC	Id	none	2/2 Id-specific T cell proliferation; no Id-specific CTL response	2/2 PD

Yi et al.	mMo-DC	Id	Il-2	2/5 Id-specific T cell proliferation; 5/5 Id-specific B cell proliferation; 4/5 Id-specific IFN-*γ*	1/3 PR; 3/5 SD; 1/5 PD

Bendandi et al.	mMo-DC	Id	none	4/4 anti-KLH response; 2/4 Th1 cytokines response	1/4 SD; 3/4 PD

Lacy et al.	APC8020 (Mylovenge)	Id	none	None reported	6/26 CR; 2/26 PR; 19/27 SD overall survival: 5.3 years of followup for alive patients

Lacy et al.	CD40 L-DCs	Id	KLH	9/9 Id-specific IFN-*γ*; 5/9 Id-specific CTL response; 8/9 anti-KLH response	6/9 SD; 3/9 slowly PD 4/6 continue SD after 5 years

Rosenblatt et al.	DC/tumor fusion		GM-CSF	11/15 CD4 and CD8 response with autologous myeloma cells; 5/5 tested anti-MUC1 response	11/16 SD (3/11 > 1 years SD; 8/11 2.5–5 months SD)

Rollig et al.	mMo-DC	Id	KLH	5/9 Id-specific T cell proliferation; 8/9 Id-specific cytokines response	3/9 M protein decrease; 5/9 M protein stable

DC: dendritic cell; TA: tumor antigen; imDC: immature DC; Mo-DC: monocyte-derived DC; Id: idiotype; mMo-DC: mature Mo-DC; KLH: keyhole limpet hemocyanin; CTL: cytotoxic T lymphocyte; PD: progressive disease; PR: partial response; SD: stable disease; CR: complete response.
